# Microbial Ecology of Artisanal Feta and Kefalograviera Cheeses, Part I: Bacterial Community and Its Functional Characteristics with Focus on Lactic Acid Bacteria as Determined by Culture-Dependent Methods and Phenotype Microarrays

**DOI:** 10.3390/microorganisms10010161

**Published:** 2022-01-13

**Authors:** Markella Tsigkrimani, Magdalini Bakogianni, Spiros Paramithiotis, Loulouda Bosnea, Eleni Pappa, Eleftherios H. Drosinos, Panagiotis N. Skandamis, Marios Mataragas

**Affiliations:** 1 Department of Food Science and Human Nutrition, Agricultural University of Athens, 75 Iera Odos Str., 11855 Athens, Greece; marktsigr@aua.gr (M.T.); magdabakogianni17@gmail.com (M.B.); sdp@aua.gr (S.P.); ehd@aua.gr (E.H.D.); pskan@aua.gr (P.N.S.); 2 Department of Dairy Research, Institute of Technology of Agricultural Products, Hellenic Agricultural Organization “DIMITRA”, 3 Ethnikis Antistaseos Str., 45221 Ioannina, Greece; louloudabosnea@gmail.com (L.B.); pappa.eleni@yahoo.gr (E.P.)

**Keywords:** Feta, Kefalograviera, Biolog, *Enterococcus faecium*, *Lactiplantibacillus plantarum*, *Weissella paramesenteroides*

## Abstract

Artisanal cheesemaking is still performed using practices and conditions derived from tradition. Feta and Kefalograviera cheeses are very popular in Greece and have met worldwide commercial success. However, there is a lack of knowledge regarding their lactic acid microecosystem composition and species dynamics during ripening. Thus, the aim of the present study was to assess the microecosystem as well as the autochthonous lactic acid microbiota during the ripening of artisanal Feta and Kefalograviera cheeses. For that purpose, raw sheep’s milk intended for cheesemaking, as well as Feta and Kefalograviera cheeses during early and late ripening were analyzed, and the lactic acid microbiota was identified using the classical phenotypic approach, clustering with PCR-RAPD and identification with sequencing of the 16S-rRNA gene, as well as with the Biolog GEN III microplates. In addition, the functional properties of the bacterial community were evaluated using the Biolog EcoPlates, which consists of 31 different carbon sources. In general, concordance between the techniques used was achieved. The most frequently isolated species from raw sheep’s milk were *Enteroroccus faecium*, *Lactiplantibacillus plantarum* and *Pediococcus pentosaceus*. The microecosystem of Feta cheese in the early ripening stage was dominated by *Lp. plantarum* and *E. faecium*, whereas, in late ripening, the microecosystem was dominated by *Weissella paramesenteroides*. The microecosystem of Kefalograviera cheese in the early ripening stage was dominated by *Levilactobacillus brevis* and *E. faecium*, and in late ripening by *W. paramesenteroides* and *E. faecium*. Finally, Carbohydrates was the main carbon source category that metabolized by all microbial communities, but the extent of their utilization was varied. Kefalograviera samples, especially at early ripening, demonstrated higher metabolic activity compared to Feta cheese. However, dominating species within microbial communities of the cheese samples were not significantly different.

## 1. Introduction

Milk and dairy products have been an essential part of human nutrition since antiquity. Indeed, their consumption is mentioned in Homer’s *Odyssey* and utensils used for their preparation, ripening and storage have been recovered from tombs of the pharaonic period [1].

The microecosystem of raw milk and dairy products have been in the epicenter of intensive research over the last decades. The complexity and importance of raw milk microbiota has been recently underlined by Quigley et al. [2]. It is widely recognized that the microbiota of milk derived from healthy animals is dominated by lactic acid bacteria (LAB), which in the case of sheep’s milk mostly belong to the former-*Lactobacillus*, the *Lactococcus* and the *Leuconostoc* genera [2]. This microbiota is particularly important for the production of fermented dairy products, the consumption of which has been correlated with a series of health benefits including the improvement of gastrointestinal, cardiovascular and bone health and the decrease of obesity and related comorbidities risks [3].

The wealth of Greek traditional dairy products, particularly cheeses, has been adequately highlighted [4], and is also depicted by the number of cheeses (21) that have been awarded the Protected Designated of Origin (PDO) status. Among them, Feta and Kefalograviera are considered the most popular and are the ones that have met worldwide commercial success. The microecosystem development during Feta cheese’s traditional manufacture and ripening has been studied to some extent [5,6,7,8]. Although prevalence of LAB was verified in all cases, differences at species level were evident and may be assigned to differences in the microbiological and physicochemical characteristics of the raw materials, as well as the manufacturing and ripening conditions and practices. On the contrary, no data are currently available regarding the microecosystem composition dynamics during the manufacturing and ripening of Kefalograviera cheese.

Epirus is a mountainous region of Northwestern Greece that has been included in the Less Favored Areas, as defined by Dir.75/268/EEC [9]. In Epirus and its neighboring region of West Macedonia, sheep and goat farming, as well as dairy products manufacturing, is still widely carried out by small-size family-based entrepreneurships [10]. A wide variety of dairy products are still produced using traditional knowledge and practices, in many cases without the aid of starter cultures, among which are the highly appreciated Feta and Kefalograviera cheeses. Despite the significance for the local economy, no studies are currently available regarding the microbial ecology and functional characteristics of artisanal Feta and Kefalograviera cheeses. Therefore, the composition of the lactic acid microecosystem, as well as the dynamics of its members during ripening, are unknown. Thus, the aim of the present study was to assess the autochthonous lactic acid microbiota composition and dynamics during the ripening of the Feta and Kefalograviera cheeses. 

## 2. Materials and Methods

### 2.1. Sampling

Samples (*n* = 5) of raw sheep’s milk were collected from different local producers in the Epirus region during the morning from refrigerated bulk tanks under aseptic conditions. Feta (*n* = 2) and Kefalograviera (*n* = 2) cheese samples were collected from Theodoriana (Arta, Epirus) and Samarina (Grevena, West Macedonia), respectively, from local producers, manufactured without the use of starter cultures, except for the rennet addition. Cheese samples were gathered at early (*n* = 2; one sample for each cheese) and late (*n* = 2; one sample for each cheese) ripening during the summer; within the same time period, the raw sheep’s milk was also sampled. All products were transferred to the laboratory at 4 °C and stored for 24 h under the same conditions for analysis.

### 2.2. Physicochemical Characterization

The Feta and Kefalograviera samples were analyzed for the following parameters: pH, moisture, fat, protein, lactose, and sodium chloride (all parameters are expressed in % except for pH). Cheeses were also examined for lipolysis (expressed in Acid Degree Value—ADV) [11] and proteolysis (the absorbance of total nitrogen soluble taken after cheese pretreatment with 12% of trichloroacetic acid—TCA) [12]. Raw sheep’s milk samples were characterized for pH, fat, protein, lactose, and non-fat solids (all parameters are expressed in % except for pH). The pH was measured by immersing a pH-meter (WTW, Weilheim, Germany) in a homogenized solution of cheese sample (10 g) with distilled water (90 mL); milk samples were used without dilution. The determination of moisture, fat, protein, lactose, and sodium chloride of cheese samples was carried out on a FoodScan (FOSS, Hilleroed, Denmark) while fat, protein, lactose, and non-fat solids of milk samples were carried out on MilkoScan (FOSS), according to manufacturers’ instructions.

### 2.3. Microbiological Analyses

A portion of 10 g of each cheese sample was homogenized with 2% (*w*/*v*) sterile sodium citrate solution (Sigma-Aldrich, St. Louis, MO, USA) (90 mL) using a stomacher apparatus (Interscience for microbiology, Saint Nom la Brétèche, France). Raw milk samples were not diluted. For the enumeration of lactic acid microbiota, decimal serial dilutions in sterile saline were spread on De Man, Rogosa, Sharpe agar (MRS) (Condalab, Madrid, Spain), Lactic Bacteria Differential agar (HiMedia Laboratories, Mumbai, India), and HiCrome Nickels and Leesment agar (HiMedia Laboratories); incubation took place at 30 °C for 72 h, 35 °C for 24–48 h, and 30 °C for 48 h, respectively. From each sample, all colonies present in the final dilution were purified by successive subculturing in MRS broth (Condalab). Purified isolates were stored at −20 °C in Nutrient Broth (LAB M, Lancashire, UK) supplemented with 30% glycerol (Applichem, Darmstadt, Germany). *Staphylococcus aureus* counts, as well as detection and enumeration of *Escherichia coli* O157:H7, *Listeria monocytogenes* and *Salmonella* spp. were also performed [13,14].

### 2.4. Lactic Acid Microbiota Identification

#### 2.4.1. Classical Identification 

Phenotypic identification of the LAB isolates was performed according to the second edition of the Bergey’s Manual of Systematic Bacteriology [15,16,17,18,19]. It included the microscopic examination of morphological characteristics, Gram stain, the ability to produce CO_2_ from glucose, growth in the presence of 4.0 and 6.5% NaCl, growth at 4, 10, 15, 35, 37 and 45 °C, as well as the ability to ferment a range of carbohydrates (L-arabinose, cellobiose, D-galactose, D-glucose, lactose, maltose, d-mannitol, melibiose, raffinose, D-ribose, sorbitol, sucrose, a-trehalose and D-xylose).

#### 2.4.2. Molecular Identification

Extraction of DNA was performed according to Doulgeraki et al. [20]. Genotypic proximity of the isolates was evaluated through PCR-RAPD using M13 as primer [21]. PCR amplicons were separated by electrophoresis in 1.5% agarose gel in 1.0× Tris Acetate EDTA (TAE) at 100 V for 1.5 h and visualized by ethidium bromide staining. Gels were scanned with GelDoc system (BioRad, Hercules, CA, USA). Conversion, normalization and further analysis was performed with the Bionumerics software (Applied Maths NV, Sint-Martens-Latem, Belgium) applying the Dice coefficient and UPGMA cluster analysis. Identification at species level was performed through sequencing of the V1–V3 region of 16S-rRNA gene, in one to three representative strains from each cluster. Species-specific PCR was also applied to the members of the *Lactiplantibacillus plantarum* group in order to differentiate them more reliably [22].

#### 2.4.3. Biolog Microbial Identification with GEN III Microplates

One hundred and eleven pure cultures of representatives from each group of lactic acid bacteria (LAB), stored at −20 °C with 30% glycerol as cryoprotectant, were resuscitated twice in a 15 mL conical sterile centrifuge tube (Corning^®^, Sigma-Aldrich) containing 10 mL of MRS broth (Condalab) (1% inoculum) and incubated at 30 °C for 18–24 h. During the final resuscitation, the cells were allowed to reach the mid-exponential growth phase (OD600 nm ~0.5) and collected. The microbial cells were harvested (10,000× *g* for 15 min at 4 °C) (Eppendorf, Hamburg, Germany) and the pellet was washed twice with sterile Ringer solution of ¼ strength (Sigma-Aldrich) before its final dilution in 1 mL of IF-C fluid (Biolog Inc., Hayward, CA, USA) to acquire a homogenous cell suspension without clumps [23]. The protocol C1, as described by the manufacturer (Biolog Microbial Identification System), was followed which is suitable for the identification of LAB: An appropriate amount of cell suspension was carefully titrated in an inoculating IF-C fluid until the desired transmittance (cell density of 95–98% transmittance) is reached, as measured by the Biolog Turbidimeter. Before microbial identification begins, the turbidimeter readings were blanked (i.e., 100% transmittance) using an IF tube filled with water, and then readings were validated using the 85% T standard (Biolog). Finally, the turbidimeter reading was re-blanked using a fresh IF-C fluid [23]. To obtain an accurate as possible cell turbidity reading when a cell suspension was added, the inoculated IF-C fluid was gently turned upside-down three times, and afterwards the tube was left still for a few seconds to allow air bubbles’ dispersion. Within 20–30 min of the IF-C fluid preparation, an aliquot of 100 μL per well was dispensed in a 96-well GEN III microplate (Biolog). The plates were incubated at 33 °C, as recommended by the manufacturer, because this temperature has been used to develop the GEN III database based on which microbial identification was performed (GEN III v2.8.0 database). At a specific time point (22 h) of incubation, and only if the A10 well was positive, the results were recorded by visual inspection of the microplates (the color density of the wells in the columns 1 to 9 were compared to the A1 negative control well which should not have a noticeable purple color; positive reaction, light/dark purple color; negative reaction, no color or visually resembling the color of the A1 well; and borderline, extremely faint purple color or when there is an uncertainty regarding the color interpretation; the color density of the wells in the columns 10 to 12 were compared to the A10 positive control well which should have a noticeable purple color; positive reaction, color similar to the A10 well; negative reaction, no color or extremely faint purple color or less than half of the color of the A10 well; and borderline, when there is an uncertainty regarding the color interpretation) and manually entered in the MicroLogM v5.2.01 software. If “no identification” was provided by the software, then the microplates were further incubated up to 40–48 h and read again. An identification at species level is specified by the algorithm when the probability that the identification made is correct exceeds the value of 0.5, otherwise the system proposes the extension of the incubation. If microbial strains continued to be unidentified after 48 h then they were registered as having “no identification” [23].

To evaluate the community-level physiological profiling (CLPP) of the microbial community during the ripening of Greek Feta and Kefalograviera cheeses the EcoPlates (Biolog Inc.), containing 31 carbon sources in triplicate (technical replicates) plus a negative control, were used. Samples were taken from early and late ripening stages and analysis was performed in duplicate (biological replicates), i.e., the metabolism of each carbon source was evaluated six times at each ripening phase. An amount of 10 g of cheese sample was homogenized in a BagFilterP of 400 mL (Interscience for microbiology) with 90 mL of a 2% (*w*/*v*) sterile sodium citrate solution (Sigma-Aldrich) for 2 min at normal speed using a BagMixer 400CC stomacher (Interscience for microbiology) and 45 mL of the filtration was centrifuged in a 50 mL conical sterile centrifuge tube (Corning) at 10,000× *g* for 15 min at 4 °C. The resulted pellet was washed twice with sterile Ringer solution of ¼ strength (Sigma-Aldrich), diluted in a final concentration of 1:1000, and aliquots of 150 μL per well were dispensed in a 96-well EcoPlate [24]. Incubation of the microplates was performed at 30 °C, and absorbance was measured at 590 nm and 750 nm at constant time intervals (0, 18, 24, 42, 48, 66, 72, 91, 98, 115, 123, 138, 145, 162, and 168 h) using a microplate spectrophotometer (Epoch, BioTek, Winooski, VT, USA). Data pre-processing and analysis (Average Well Color Development—AWCD, Richness Index—S, Shannon-Wiener Index—H, Shannon Evenness Index—E, and Simpson Index—D) was carried out based on the work of Li et al. [25]. Principal Component Analysis (PCA) was performed on the mean pre-processed absorbance values of the 31 carbon sources, normalized towards the mean AWCD value of the plate [26]. Data analysis (diversity indices, fermentation of different groups of carbon sources and PCA) was completed based on the incubation time point (h) estimated separately for each sample (cheese, ripening stage, and replicate) and derived from the AWCD curve of the respective plate, i.e., the time that corresponds to the upper inflection point of the sigmoidal AWCD curve. PCA was conducted with the Past v4.08 software [27]. Graphs and statistical analysis (two-way ANOVA and corrected *p*-values to account for multiple comparisons using the Tukey statistical hypothesis testing with family-wise alpha threshold and confidence interval of 0.05 and 95%, respectively) were made with the GraphPad Prism v9.3 (GraphPad Software, San Diego, CA, USA).

## 3. Results

### 3.1. Physicochemical and Microbiological Characterization

The physicochemical characteristics of the raw sheep samples assessed in the present study were comparable and had only marginal differences. On average, the pH value was 6.8, while fat, protein, lactose and non-fat solids were 6.05, 5.4, 4.6 and 10.7%, respectively. Total aerobic mesophilic counts ranged from 3.15 to 4.82 log CFU/g. The *Staphylococcus aureus* population was below the enumeration limit (2 log CFU/mL) and the absence of *E. coli* O157:H7, *L. monocytogenes* and *Salmonella* sp. was noticed. In Table 1 the physicochemical and microbiological characteristics of the Feta and Kefalograviera cheeses in early and late ripening, are shown. In both products, fat, lactose and protein hydrolysis were constant. In the case of Feta cheese, a reduction in moisture, pH value, fat hydrolysis and MRS counts was observed. On the contrary, NaCl and protein content increased. Regarding Kefalograviera, a small increase was observed in moisture and a small decrease in pH value, NaCl and protein content. Finally, fat hydrolysis and MRS counts were increased during later ripening. As in the case of raw sheep’s milk, the *S. aureus* population was below the enumeration limit and the absence of *E. coli* O157:H7, *L. monocytogenes* and *Salmonella* sp. was observed. 

### 3.2. Lactic Acid Microbiota Identification

#### 3.2.1. Classical Identification

A total of 189 bacterial isolates were obtained from the nine samples assessed. Their classification was performed by the evaluation of their phenotypic and genotypic characteristics. In Table S1 the biochemical characteristics of the isolates is presented. On the basis of the cell morphology, the isolates were differentiated into cocci (91 isolates) and bacilli (98 isolates). Cocci were further subdivided into 20 groups of isolates that were homofermentative, and four groups that were able to produce CO_2_ from glucose. On the other hand, no homofermentative bacilli were isolated and the heterofermentative ones were further subdivided into 13 groups. Based on the Bergey’s Manual of Systematic Bacteriology, groups 1–8 were identified as *Enterococcus faecium*, group 9 as *E. faecalis*, group 10 as *Lactococcus lactis*, groups 11–19 as *Pediococcus pentosaceus*, groups 20–23 as *Leuconostoc mesenteroides*, group 24 as *Lactiplantibacillus pentosus*, group 25 as *Latilactobacillus curvatus*, groups 26–27 as *Lp. plantarum*, groups 28–29 as *Levilactobacillus brevis* and groups 30–36 as *Weissella paramesenteroides*.

#### 3.2.2. Molecular Identification

**Figure 1:**
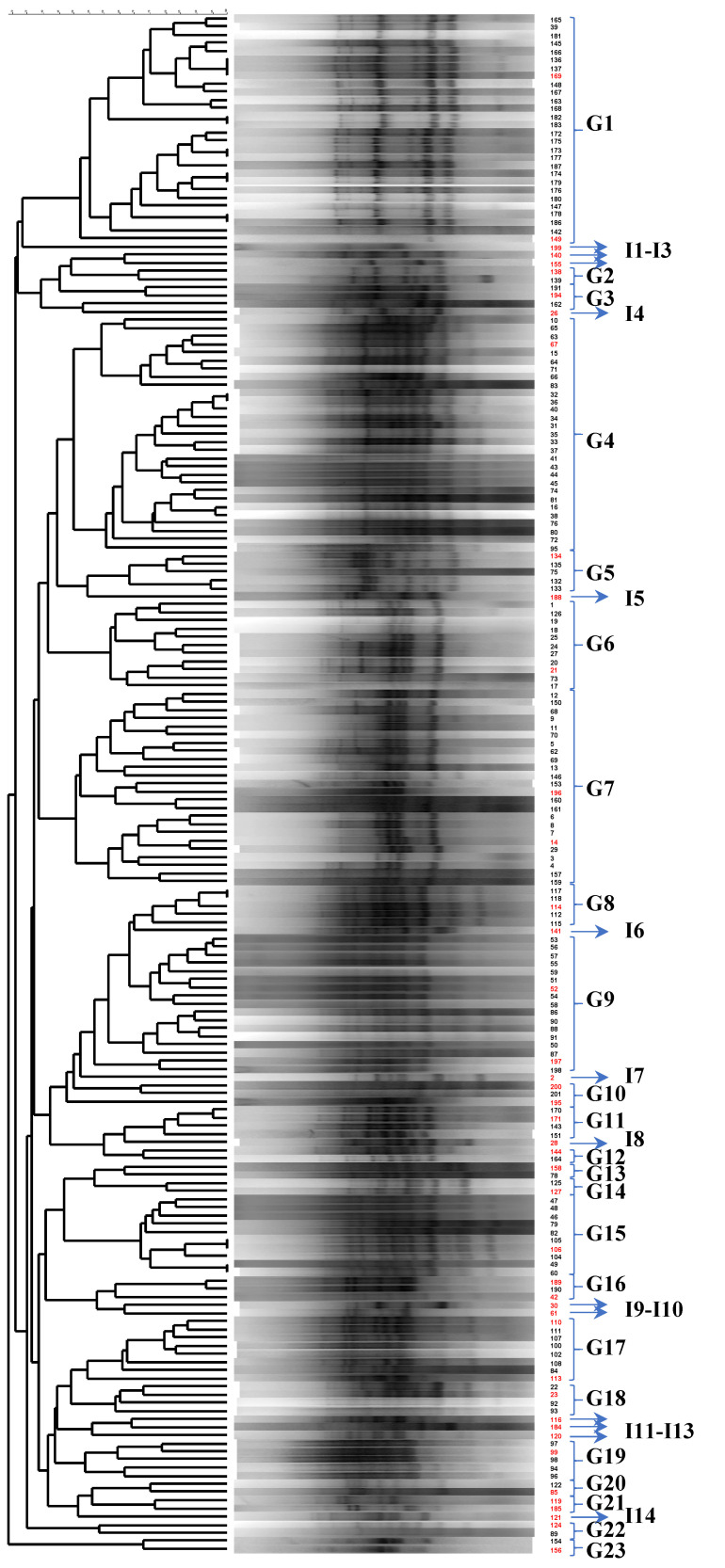
Cluster analysis of PCR-RAPD patterns of bacterial isolates, obtained from nine dairy samples. Distance is indicated by the mean correlation coefficient [r (%)] and clustering was performed by UPGMA analysis. The representative strains selected for 16S rRNA gene sequencing are in red.

The application of RAPD-PCR resulted in the separation of the isolates into 23 groups, designated G1 to G23 (Figure 1). In addition, 14 isolates failed to cluster to one of the aforementioned groups; these isolates were designated I1 to I14. The latter isolates, as well as representative ones from each group, had their 16S rRNA gene sequenced. The alignment of these sequences with the ones present is in the National Center for Biotechnology information (NCBI) database, using the Basic Local Alignment Search Tool (BLAST), revealed the taxonomic affiliation of the isolates.

As a result, G1, G3 and I3 were assigned to *W. paramesenteroides*, G14-G17, G21, I1, I5, I10 and I12 to *P. pentosaceus*, G2, I2, I6, I7 and I9 to *Ln. mesenteroides*, G6, G7, G19, G23, I4, I11, I13 and I14 to *E. faecium*, G4 to *Lp. plantarum*, G5 and G20 to *Lc. lactis*, G8–G10 and G18 to *Lv. brevis*, G11 and G12 to *Lt. curvatus*, G13 and I8 to *E. faecalis*, and G22 to *Lp. pentosus*. The taxonomic affiliation of the isolates belonging to the *Lp. plantarum*-group was successfully verified by species-specific PCR, as described in Materials and Methods. Phenotypic and genotypic identification of the isolates were in absolute concordance. 

#### 3.2.3. Biolog Microbial Identification with GEN III Microplates

**Figure 2:**
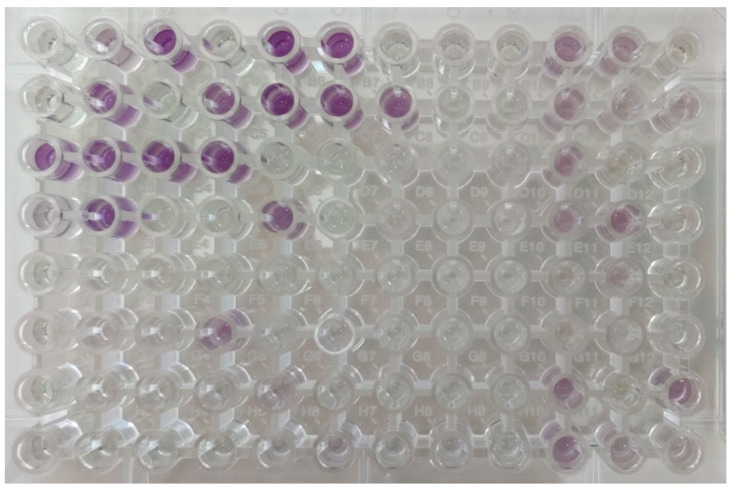
Representative GEN III microplate showing the metabolic fingerprint (color development after 22 h of incubation at 33 °C) of a microorganism based on which its identification is made.

GEN III microplates contain a tetrazolium dye that changes color because of the substrate metabolism, providing a metabolic fingerprint of the microorganism. Identification of the microbial species is performed by the Biolog database, based on the purple color pattern developed on the GEN III microplate during incubation (Figure 2). In Feta and Kefalograviera, the main LAB genera identified were the (*n* = 111): *E. faecium* group (28.8%), *Lp. plantarum* subsp. *plantarum* (11.7%), *P. acidilactici* group (11.7%), *Weissella* group (15.3%), *Lentilactobacillus buchneri* group (13.5%), *Lc. lactis* group (8.1%), *Lacticaseibacillus casei* (4.5%) *Ln. lactis* (0.9%), *Carnobacterium gallinarum* (0.9%), *Lc. garvieae* (0.9%), *Lactobacillus helveticus* (0.9%), *Liquorilactobacillus mali* (0.9%), and ‘no identification’ (1.8%). By comparing these results with those obtained from the molecular techniques applied to the identification of the respective isolates, the concordance between the biochemical and molecular characterization at genus or species level was 71.6%, as the following species were identified by the 16S rRNA sequencing: *E. faecium* (28.4%), *Lp. plantarum* (19.3%), *P. pentosaceus* (9.2%), *W. paramesenteroides* (22.9%), *Lv. brevis* (14.7%), *Lt. curvatus* (4.6%), and *Ln. mesenteroides* (0.9%).

### 3.3. Lactic Acid Bacteria Microecosystem of Raw Sheep’s Milk, Feta and Kefalograviera

**Figure 3:**
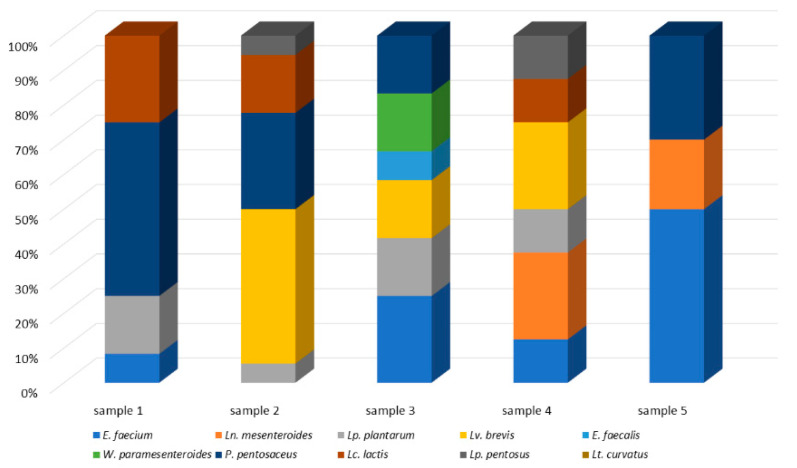
The microecosystem of the five sheep’s milk samples that were analyzed in the present study.

The microecosystem of the five raw sheep’s milk samples analyzed consisted of a consortium of three to six LAB (Figure 3). The most frequently isolated species were *E. faecium*, *Lp. plantarum* and *P. pentosaceus*, which were isolated from four samples each. On the contrary, the least common were *E. faecalis* and *W. paramesenteroides*, that were recovered by only one milk sample. Based on the number of isolates per sample, the dominance of *P. pentosaceus*, *Lv. brevis* and *E. faecium* was indicated for samples 1, 2 and 5, respectively. On the contrary, no such was indicated for samples 3 and 4.

**Figure 4:**
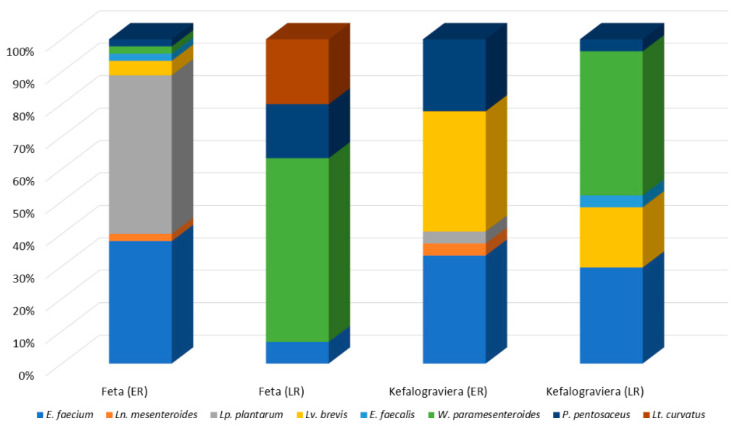
The microecosystem of Feta and Kefalograviera cheese in early (ER) and late (LT) ripening.

The microecosystem of Feta and Kefalograviera cheese in early and late ripening are exhibited in Figure 4. The microecosystem of Feta cheese in the early ripening stage was dominated by *Lp. plantarum* and *E. faecium*; the presence of *Ln. mesenteroides*, *Lv. brevis*, *E. faecalis*, *W. paramesenteroides* and *P. pentosaceus* was also observed. However, in late ripening, the microecosystem was dominated by *W. paramesenteroides* with *E. faecium*, *P. pentosaceus* and *Lt. curvatus* forming the secondary microbiota. The microecosystem of Kefalograviera cheese in the early ripening stage was dominated by *Lv. brevis* and *E. faecium*; *Ln. mesenteroides*, *P. pentosaceus* and *Lp. plantarum* were also recovered. 

As in the case of Feta cheese, in late ripening, the microecosystem composition was altered and dominated by *W. paramesenteroides* and *E. faecium*, with *Lv. brevis*, *P. pentosaceus* and *E. faecalis* being also present.

### 3.4. Biolog Microbial Community Assay with EcoPlates

**Figure 5:**
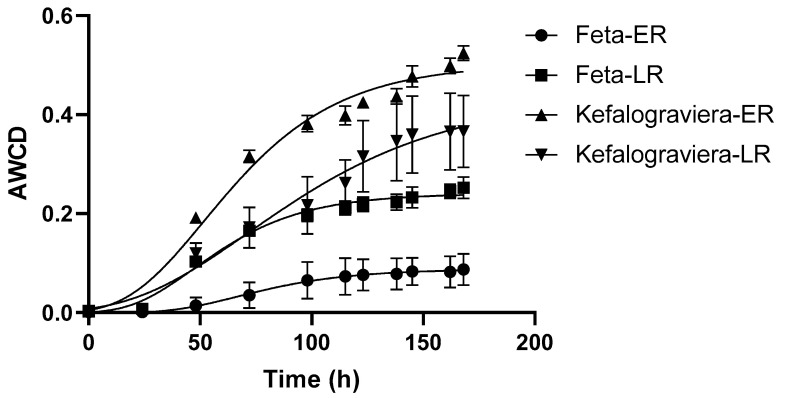
Average Well Color Development of the Biolog EcoPlate incubated at 30 °C for 168 h, reflecting the metabolic activity of the microbial community of Feta and Kefalograviera cheese samples during their ripening. ER, early ripening; LR, late ripening; sigmoidal solid line, predicted values drawn by fitting the Gompertz equation to the observed values with non-linear regression; solid symbols, observed data expressed as mean values of six replicates (three technical × two biological) along with error bars (standard deviation).

The Average Well Color Development (AWCD) of the Biolog EcoPlate was exponentially increased after an initial lag phase, and reached a plateau (Figure 5). Therefore, the microbial community of the Feta and Kefalograviera was able to metabolize the different carbon sources during the ripening of the products, but this activity was varied between the cheese samples and time of ripening within the same cheese, as suggested by the different sigmoidal AWCD curves [28]. The upper inflection point of the sigmoidal AWCD curve, that corresponds to the approximate time at which the stationary phase begins, was used for data analysis (diversity indices of metabolic activity, fermentation of different groups of carbon sources, and PCA). Therefore, day 5 for Feta-ER (early ripening) and Feta-LR (late ripening) (i.e., 115 h; an exception was the first replicate of the Feta-ER sample for which the incubation time of 145 h was applied), and day 6 for Kefalograviera (i.e., 145 h for Kefalograviera-ER or 138 h for Kefalograviera-LR) was estimated as the most appropriate incubation time according to the Gompertz equation fitting (Figure 5).

**Figure 6:**
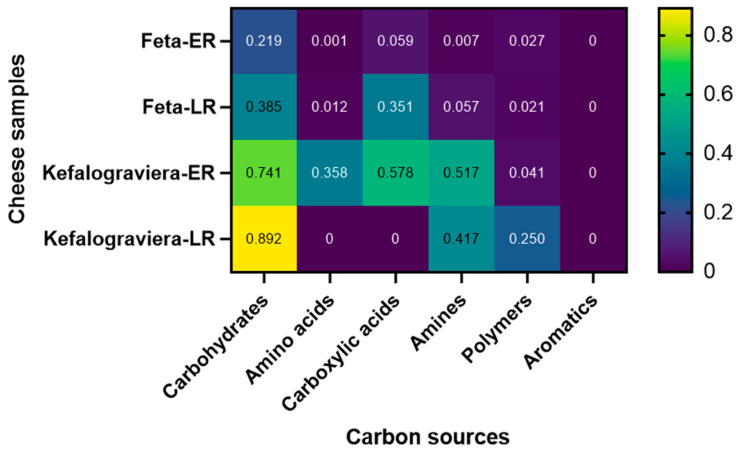
Heat map of the fermentation of different groups of carbon sources by the microbial community of each cheese sample. Each square of the heat map displays the average absorbance value estimated from all carbon sources of each group and all replicates (three technical × two biological). Color scale indicates the extent of carbon source utilization (dark purple to yellow, low to high).

**Figure 7:**
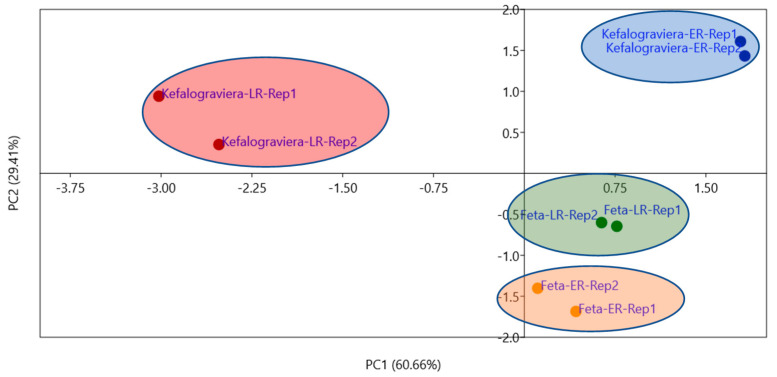
Principal Component Analysis (PCA) of cheese samples. The PC1 explained the 60.66% and PC2 the 29.41% of the total data variability. The location of the data is indicative of the specific metabolic activity of the microbial community of each cheese sample.

Carbohydrates was the main carbon source category that was metabolized byall microbial communities, but the extent of their utilization was varied significantly (*p* < 0.05) between Feta and Kefalograviera, as well as between early and late ripening. Feta-ER showed the lowest value, while Kefalograviera-LR showed the highest, i.e., Feta-ER < Feta-LR < Kefalograviera-ER < Kefalograviera-LR, indicating increased carbohydrates utilization with increased ripening time (Figure 6). On the other side, the group of Aromatics were not utilized by the microbial community of any cheese sample. The Kefalograviera-ER sample demonstrated the highest metabolic activity, in which the microorganisms were capable of fermenting four different types of carbon sources, namely carbohydrates, Amino acids, Carboxylic acids, and Amines. Finally, new carbon sources not initially utilized were fermented at the late stages of ripening, such as Carboxylic acids (Feta-LR) and Polymers (Kefalograviera-LR). PCA clearly distinguished the cheese samples based on the type and time of ripening (Figure 7). The location of the data is suggestive of the different metabolic characteristics of microbial communities [29]. Loading scores of the carbon sources showed that Carbohydrates and Carboxylic acids had a larger effect on PC1, whereas Carbohydrates, Carboxylic acids, Amino acids, and Amines had a larger effect on PC2. The Feta cheese samples were located in the bottom of the PCA plot, meaning a lower utilization of the carbon sources compared to the Kefalograviera cheese, which showed positive PC2 values, i.e., a higher utilization of the different types of carbon sources.

Substrate richness (S) and utilization pattern (H) were significantly (*p* < 0.05) increased in Feta over time, while the opposite was true in Kefalograviera (Table 2). Early stages of ripening in Feta displayed the lowest substrate utilization and richness values, and the highest values were found in Kefalograviera during the first days of ripening. The higher the S and H indices, the larger the metabolic diversity of the microbial community. However, Simpson Index—D and Shannon Evenness Index—E between the samples were not significantly different, meaning that there were common species between the samples, such as *E. faecium* and *P. pentosaceus* followed by *Lv. brevis* and *W. paramesenteroides* (Figure 4).

## 4. Discussion

The development of starter cultures has allowed the production of standardized fermented products with tailored technological, sensorial and functional attributes. This is also the case for dairy products. However, artisanal cheesemaking is still performed using practices and conditions derived from tradition. Feta and Kefalograviera cheeses are very popular in Greece; however, a lack of knowledge regarding their lactic acid microecosystem composition and species dynamics during ripening is evident.

The physicochemical characteristics in the early and late ripening of Kefalograviera cheese are in accordance with the ones presented by Katsiari et al. [30,31,32]. Similarly, the physicochemical characteristics in the early and late ripening of Feta cheese [7,33]. Marginal differences may be attributed to differences in manufacturing conditions and practices. 

The lactic acid microbiota of the raw sheep’s milk samples assessed in the present study are generally in concordance with those reported in the literature. The frequent occurrence of *Lp. plantarum* in raw sheep’s milk has been reported by Medina et al. [34], Patil et al. [35] and Quintana et al. [36]. In addition, the presence of *W. paramesenteroides*, *Ln. mesenteroides*, *Lv. brevis*, *Lc. lactis*, *Lp. pentosus* (as part of the *Lp. plantarum*-group), and *Lt. curvatus* has also been reported [34,36]. The frequent presence of *P. pentosaceus* and *E. faecium* may be attributed to their omnipresence in the livestock environment [36], especially regarding the latter. It may also indicate a hygienically compromised milking environment [34].

Species succession is a very common attribute of the lactic acid microbiota of spontaneously fermented products. Indeed, it has been reported in a variety of fermented vegetable, fruit, meat and dairy products [13,37,38,39]. In general, this succession is commonly attributed to the level of adaptation to a specific niche, in terms of ability to utilize available nutrient resources and tolerate metabolite accumulation. The species dynamics and succession during the ripening of artisanally made Feta cheese has been studied to some extent. It seems that members of the *Lp. plantarum* group are very often isolated during ripening; on the other hand, *E. faecium* is not as frequently recovered [5,6,7,8]. In addition, it seems that both species are gradually replaced by other species during ripening. This was also the case in the present study, *Lp. plantarum* co-dominated with *E. faecium* during early ripening and then, being replaced, only the latter formed a small percentage of the microecosystem during late ripening. This species succession seems to favor species such as *Lv. brevis* and some species of the *Leuconostoc* genus [7,8], in which the frequency of isolation seems to increase with ripening. In the present study, *Lv. brevis* and *Ln. mesenteroides* were only detected during early ripening and not during late ripening. On the contrary, *W. paramesenteroides* dominated during late ripening, in the presence of *P. pentosaceus*, *Lt. curvatus* and *E. faecium*. Occurrence of the latter species during Feta manufacturing has been reported [7,8,40]. Occurrence of *W. paramesenteroides* in a wide variety of fermented foods, including dairy products, has been reported [41,42,43,44,45,46,47,48,49,50,51,52,53,54], indicating a large metabolic capacity [55], which may possibly justify its dominance during the late ripening of Feta cheese. 

The lactic acid microbiota of Kefalograviera cheese, and the species dynamics and succession during ripening have not been previously assessed. The population of *E. faecium*, *Lv. brevis* and *P. pentosaceus* that dominates early ripening seems to decrease during late ripening and is substituted by *W. paramesenteroides*. This was also the fate of *Ln. mesenteroides* and *Lp. plantarum* that were only present during early ripening, as well as *E. faecalis* that was detected only in late ripening.

Sole-carbon source utilization (Biolog GEN III microplates) can be employed for rapid identification of the LAB community of the ripened Feta and Kefalograviera cheeses. Microbial identification was in an acceptable range and, therefore, this technique is useful for fast screening of the microbial community of fermented and non-fermented foods, but preferably in conjunction with other molecular techniques for obtaining the most accurate results at species level due to discrepancies observed in the microbial identification. Although the concordance between biochemical tests and molecular identification was not perfect, this is not surprising as the microbial identification which relies on the use of biochemical tests, and specifically on the Biolog GEN III microplates, have shown values at this level [56]. In addition, Biolog EcoPlates were able to provide an overview and a rapid screening of the metabolic characteristics of cheese microbial communities, enabling the direct comparison and separation of the samples by cheese type and time of ripening. Metabolic activity is more intense in Kefalograviera during the first days of ripening, while in Feta metabolic activity is progressively increased during ripening. Furthermore, when the community-level physiological profiling (CLPP) is accompanied with the microbial identification, the investigation of the microbial community structure is facilitated.

## 5. Conclusions

Species succession is the key characteristic of Feta and Kefalograviera ripening. In both cheeses, *W. paramesenteroides* seem to play an important role, since it dominated the lactic acid microecosystem during late ripening. Despite that, the assessment of community-level physiological profiling enabled effective differentiation between the samples, highlighting the importance of production conditions, as well as accompanying microbiota.

## Figures and Tables

**Table 1:** Physicochemical and microbiological characteristics of Feta and Kefalograviera cheeses in early and late ripening.

Samples	Fat (%)	Moisture (%)	pH	NaCl (%)	Lactose (%)	Protein (%)	Fat Hydrolysis	Protein Hydrolysis	MRS Counts (log CFU/mL)
Feta									
Early ripening	25.5	51.3	5.07	2.53	0	15.85	3.9	0.764	7.02
Late ripening	26	49.3	4.25	3.4	0	16.24	2	0.702	6.60
Kefalograviera									
Early ripening	30.5	36.1	5.14	3.05	0.3	26.28	2.5	0.58	6.02
Late ripening	30	36.7	5.12	2.91	0.31	26.10	4.1	0.589	6.77

*S. aureus* population was below enumeration limit (2 log CFU/mL). Absence of *E. coli* O157:H7, *L. monocytogenes*, *Salmonella* sp. was verified.

**Table 2:** Estimated metabolic diversity indices (mean ± stdev, *n* = 6) of the cheese microbial communities.

Cheese Samples	*Richness—S*	*Shannon-Wiener—H*	*Shannon Evenness—E*	*Simpson—D*
Feta-ER	2.500 ± 0.236 ^a^	1.154 ± 0.104 ^a^	1.349 ± 0.319 ^a^	0.601 ± 0.012 ^a^
Feta-LR	8.667 ± 0.472 ^b^	2.307 ± 0.040 ^b^	1.069 ± 0.008 ^a^	0.880 ± 0.006 ^a^
Kefalograviera-ER	14.167 ± 0.235 ^c^	2.650 ± 0.003 ^b,c^	1.002 ± 0.006 ^a^	0.922 ± 0.001 ^a^
Kefalograviera-LR	7.167 ± 1.179 ^d^	1.821 ± 0.173 ^d^	0.932 ± 0.003 ^a^	0.810 ± 0.039 ^a^

Different letters within each column show significant difference at *p* < 0.05.

## Data Availability

The data presented in this study are available in the manuscript.

## Supplementary Materials

The following supporting information can be downloaded at: https://www.mdpi.com/article/10.3390/microorganisms10010161/s1, Table S1: Biochemical properties used to identify the bacterial isolates.
