# Supplementary material for: Microbial Ecology of Artisanal Feta and Kefalograviera Cheeses, Part I: Bacterial Community and Its Functional Characteristics with Focus on Lactic Acid Bacteria as Determined by Culture-Dependent Methods and Phenotype Microarrays
Source: Microorganisms. 2022 Jan 13;10(1):161. doi: 10.3390/microorganisms10010161 (PMC8780491; doi:10.3390/microorganisms10010161)
Supplement: Supplementary file 1 [file microorganisms-10-00161-s001.zip › microorganisms-1532331-supplementary.pdf]

Table S1. Biochemical properties used to identify the bacterial isolates.

Group Number	Isolate Number	Cell morphology [a]	CO_2 from glucose	Growth (%NaCl)		Growth (ºC)		Acid from												
				4	6.5	10/15 [b]	45	lactose	mannitol	sucrose	maltose	galactose	sorbitol	raffinose	ribose	cellobiose	trehalose	xylose	melibiose	L-arabinose
1	1, 3, 4, 5, 6, 7, 11, 12, 14, 19, 20, 21, 24, 25, 94, 96, 97, 98, 99	c	-	+	+	+	+	+	+	+	+	+	+	+	+	+	+	-	-	+
2	8, 9, 13, 18, 26, 27, 29	c	-	+	+	+	+	+	+	+	+	+	+	+	+	+	+	-	+	+
3	17	c	-	+	+	+	+	+	+	+	+	+	+	+	+	+	+	+	+	+
4	126, 146, 150, 153, 154, 156, 157, 159	c	-	+	+	+	-	+	+	+	+	+	+	+	+	+	+	-	-	+
5	62, 68, 69, 70, 73, 120, 121	c	-	+	+	+	-	+	+	+	+	+	+	+	+	+	+	-	+	+
6	116	c	-	+	+	+	-	+	+	+	+	+	+	+	+	+	+	+	+	+
7	160, 161,	c	-	+	+	+	-	+	+	+	+	+	-	+	+	+	+	-	+	+
8	196	c	-	+	+	+	-	+	+	-	+	+	+	+	+	+	+	+	+	+
9	28, 78, 158	c	-	+	+	+	+	+	+	+	+	+	+	-	+	+	+	-	-	-
10	75, 85, 122, 132, 133, 134, 135	c	-	+	-	+	-	+	-	-	+	+	-	-	+	-	-	-	-	-
11	42, 111, 113, 119, 125, 189	c	-	-	-	-	-	+	-	+	+	+	-	+	+	+	+	-	+	-
12	46, 47, 48, 49, 60, 61, 79, 82, 84, 110, 127, 199	c	-	-	-	-	-	+	-	+	+	+	-	+	+	+	+	+	+	-
13	100, 102	c	-	-	-	-	-	+	-	+	+	+	-	-	+	+	+	+	+	-
14	104, 107, 108,	c	-	-	-	-	-	+	-	+	+	+	-	-	+	+	+	+	-	-
15	105	c	-	-	-	-	-	+	-	+	+	+	-	+	+	+	+	+	-	-
16	106	c	-	-	-	-	-	+	-	+	+	+	-	+	+	+	+	-	-	-
17	184, 185	c	-	-	-	-	-	+	-	+	+	+	-	-	+	+	+	-	+	-
18	190	c	-	-	-	-	-	+	-	+	+	+	-	-	+	+	+	-	-	-
19	188	c	-	-	-	-	-	+	-	-	+	+	-	-	+	+	+	+	-	-
20	2	c	+	-	-	-	-	+	+	+	+	+	-	+	-	+	+	-	+	-

21	30	c	+	-	-	-	-	+	+	+	+	+	-	-	-	+	+	-	+	-
22	138, 141	c	+	-	-	-	-	+	+	+	+	+	-	+	+	+	+	+	+	-
23	139, 140	c	+	-	-	-	-	+	+	+	+	+	-	+	+	+	+	-	+	-
24	89, 124	b	+	-	-	+	-	-	+	+	-	-	-	+	+	+	-	+	+	-
25	143, 144, 151, 164, 170, 171	b	+	-	-	+	-	-	-	+	-	-	-	-	+	+	-	-	-	-
26	10, 31, 32, 33, 34, 35, 36, 65, 66, 67, 80, 81, 95	b	+	-	-	+	-	-	+	+	-	-	+	+	+	+	-	+	+	-
27	15, 16, 37, 38, 40, 41, 43, 44, 45, 63, 64, 71, 72, 74, 76, 83	b	+	-	-	+	-	-	+	+	-	-	+	+	+	+	-	-	+	-
28	22, 23, 50, 51, 52, 53, 54, 55, 56, 57, 58, 59, 86, 87, 88, 90, 91, 92, 93, 112, 114, 195, 197, 198, 200, 201	b	+	-	-	+	-	-	-	+	+	+	-	+	+	-	-	+	+	-
29	115, 117, 118	b	+	-	-	+	-	-	-	+	+	-	-	+	+	-	-	+	+	-
30	39, 136, 137, 142, 145, 149, 162, 175, 176, 177, 178, 179, 180, 181, 182	b	+	-	-	-	-	-	-	+	+	+	-	+	-	+	+	+	+	-
31	147, 148, 155, 183, 186	b	+	-	-	-	-	-	-	+	+	+	-	+	-	+	+	-	+	-
32	163, 165, 166, 167	b	+	-	-	-	-	-	-	+	+	+	-	+	-	-	+	+	+	-
33	168	b	+	-	-	-	-	-	-	+	+	+	-	+	-	-	+	-	+	-
34	169, 194	b	+	-	-	-	-	-	-	+	+	+	-	-	-	-	+	-	+	-
35	172, 173, 174	b	+	-	-	-	-	-	-	+	+	+	-	-	-	-	+	+	+	-
36	187, 191	b	+	-	-	-	-	-	-	+	+	+	-	-	-	+	+	-	+	-

[a] b: bacilli; c: cocci

[b] 10 °C for cocci; 15°C for bacilli
